# Quantum simulation of thermodynamics in an integrated quantum photonic processor

**DOI:** 10.1038/s41467-023-38413-9

**Published:** 2023-07-01

**Authors:** F. H. B. Somhorst, R. van der Meer, M. Correa Anguita, R. Schadow, H. J. Snijders, M. de Goede, B. Kassenberg, P. Venderbosch, C. Taballione, J. P. Epping, H. H. van den Vlekkert, J. Timmerhuis, J. F. F. Bulmer, J. Lugani, I. A. Walmsley, P. W. H. Pinkse, J. Eisert, N. Walk, J. J. Renema

**Affiliations:** 1 MESA+ Institute for Nanotechnology, University of Twente, P. O. box 217, 7500 AE Enschede, The Netherlands; 2 Dahlem Center for Complex Quantum Systems, Freie Universität Berlin, 14195 Berlin, Germany; 3 QuiX Quantum B.V., Hengelosestraat 500, 7521 AN Enschede, The Netherlands; 4 Quantum Engineering Technology Labs, University of Bristol, Bristol, UK; 5 Center for Sensors, Instrumentation and Cyber Physical System Engineering, IIT Delhi, New Delhi, 110 016 India; 6 Department of Physics, Imperial College London, Prince Consort Rd., London, SW7 2AZ UK; 7 Clarendon Laboratory, University of Oxford, Parks Road, Oxford, OX1 3PU UK; 8 Helmholtz-Zentrum Berlin für Materialien und Energie, 14109 Berlin, Germany; 9 Fraunhofer Heinrich Hertz Institute, 10587 Berlin, Germany

**Keywords:** Quantum simulation, Quantum information, Quantum optics

## Abstract

One of the core questions of quantum physics is how to reconcile the unitary evolution of quantum states, which is information-preserving and time-reversible, with evolution following the second law of thermodynamics, which, in general, is neither. The resolution to this paradox is to recognize that global unitary evolution of a multi-partite quantum state causes the state of local subsystems to evolve towards maximum-entropy states. In this work, we experimentally demonstrate this effect in linear quantum optics by simultaneously showing the convergence of local quantum states to a generalized Gibbs ensemble constituting a maximum-entropy state under precisely controlled conditions, while introducing an efficient certification method to demonstrate that the state retains global purity. Our quantum states are manipulated by a programmable integrated quantum photonic processor, which simulates arbitrary non-interacting Hamiltonians, demonstrating the universality of this phenomenon. Our results show the potential of photonic devices for quantum simulations involving non-Gaussian states.

## Abstract (web summary)

The emergence of relaxation in unitarily evolving systems can be seen as a paradox, but not once the distinction between local and global dynamics is considered. Here, the authors use photons in an integrated optical interferometer to show that, for a system evolving unitarily on a global level, single-mode measurements converge to those of a thermal state.

## Introduction

**Fig. 1:**
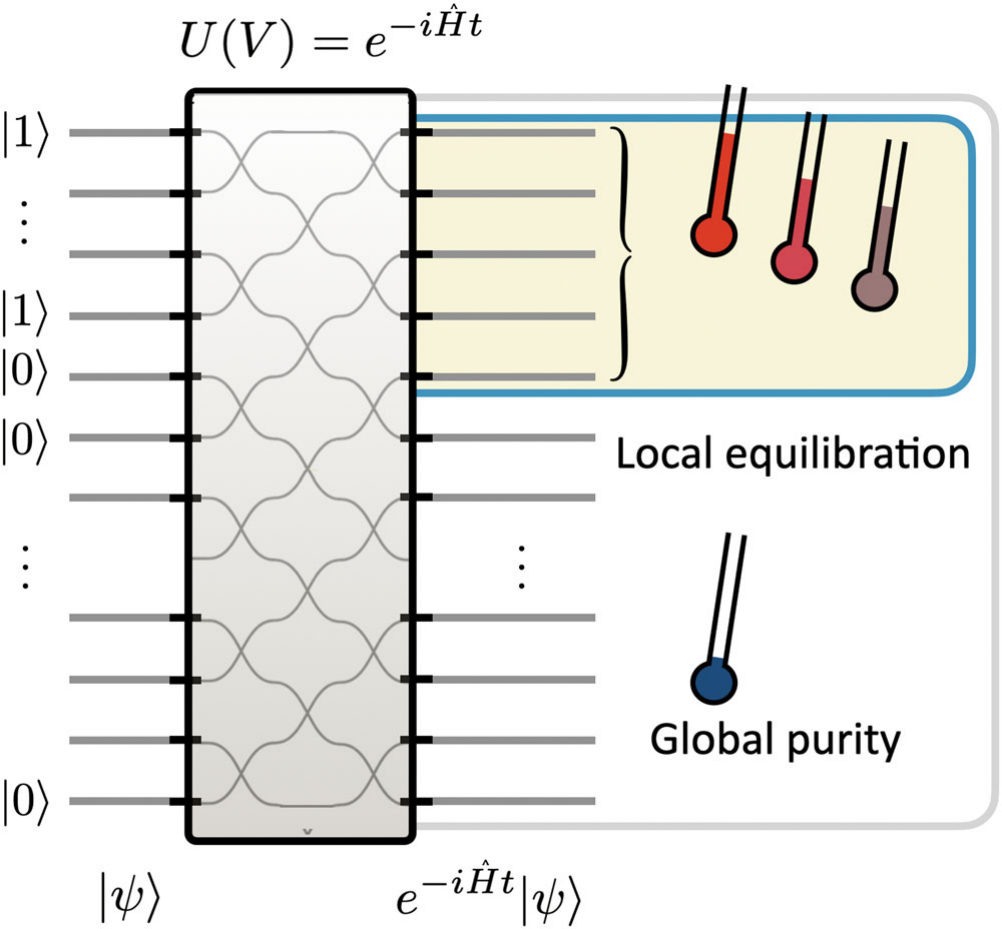
Photonic simulation of quantum equilibration. A closed, many-body quantum system, initialized in a product state and undergoing unitary evolution generated by a Hamiltonian, necessarily remains in a pure state. However, local observables may exhibit a generalized thermalization. Entanglement builds up between sub-systems until, after some time *t*_eq_, each sub-system appears to have approximately relaxed into a maximum entropy state. The paradigmatic case of a non-Gaussian bosonic state evolving under a quadratic Hamiltonian can be probed via a photonic simulation platform. A fully programmable linear optical chip can provide ‘snapshots’ of the local and global system dynamics for arbitrary times and interaction ranges by implementing the appropriate unitary $U(V)={e}^{-i\hat{Ht}}$ with *V* ∈ U(*m*) for *m* modes.

One of the long-standing puzzles of theoretical physics is how notions of statistical physics and basic quantum mechanics fit together in closed systems^1^. Statistical mechanics is concerned with probabilistic, stationary ensembles that maximize entropy under external constraints. Elementary quantum mechanics, in contrast, describes the deterministic evolution of quantum states of closed systems under a specified Hamiltonian. It has become clear^2–5^ that these seemingly contradictory premises can be resolved by making the distinction between *global* unitary dynamics and *local* relaxation (see Fig. 1). The physical mechanism is that local expectation values converge to those of statistical ensembles, while the entire closed quantum system undergoes unitary dynamics. Large-scale, closed quantum systems, therefore, appear locally thermal without the need to postulate an external heat bath. Crucially, this local equilibration behavior is believed to be ubiquitous, in the sense that one has to fine-tune the Hamiltonian in order to not observe it^5,6^.

The mechanism of local equilibration is particularly clear-cut under non-interacting quadratic bosonic Hamiltonians, such as described in linear quantum optics. If the initial state is non-Gaussian, it is expected to ‘Gaussify’ in time, i.e., to locally converge to Gaussian states that maximize the entropy given all second moments of the state^7–11^. In this case, for local Hamiltonian dynamics, it can be rigorously proven^7–9,11^ that the state converges to a so-called generalized Gibbs ensemble (GGE)^7,12–15^, i.e., a thermal ensemble under further constants of motion or conserved charges. Notwithstanding this comparably clear theoretical situation, only very recently, there has been substantial experimental progress^10,16–19^, with still not all aspects being clarified. This is primarily due to the fact that it is challenging to create sufficiently isolated experimental systems to rule out that the observed equilibration is not due to decoherence but in fact, to the desired dynamics^16,20–25^.

**Fig. 2:**
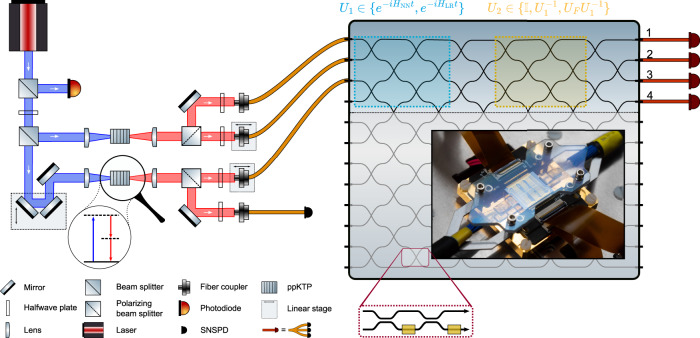
Overview of the setup. The left-hand side of the figure shows the two spontaneous parametric down-conversion (SPDC) sources based on nonlinear *periodically poled Potassium Titanyl Phosphate* (ppKTP) crystals, in which blue pump photons are spontaneously split into two red photon pairs. One of these four photons is used as a herald, and the other three are injected in the first three modes of our 12 × 12 integrated photonic programmable processor. The processor output is sent to small fiber-beam-splitter networks and superconducting nanowire single-photon detectors (SNSPDs), which act as quasi-photon number resolving detectors. In the processor, we program the unitary *U*_1_ used to simulate the temporal dynamics (the blue block). In addition, we can program a second unitary *U*_2_ for the verification process (the yellow block). The zoom-in shows a Mach–Zehnder interferometer that implements one of the programmable beam splitters. The inset shows a photograph of a fiber-connected integrated optical chip nominally identical to the one used in the experiment. Photo credit for the inset photo: Gijs van Ouwerkerk (PHIX Photonics Assembly). Photo credit for the rest of the figure: The authors.

In this work, we experimentally show universal, reversible equilibration and Gaussification using an integrated quantum photonic processor (see Fig. 2), i.e., a programmable linear optical interferometer. We use the very high degree of control available in integrated photonics to simulate, for arbitrary interaction times, a large number of randomly chosen quadratic Hamiltonians, including ones that are not restricted to nearest-neighbor coupling. We exploit the size of the optical network to implement a set of additional optical transformations that certify that the observed relaxation is due to the internal dynamics of our multi-mode quantum state, and not due to interaction with the environment, by undoing the Hamiltonian. We find that the single-mode measurements converge to those of a thermal state with a temperature corresponding to the mean photon number, while the overall time evolution can be undone, which certifies universal, reversible Gaussification. These results exemplify the advantages of photonics as a platform for quantum simulation^26–32^, namely good scaling of decoherence with system size, a high degree of experimental control, and the rapid growth in achievable quantum systems, both measured in the number of optical modes and in the number of photons. The fact that photonic quantum interference without explicit photon–photon interactions carries computational hardness, as demonstrated by the hardness of boson sampling^33–35^, shows that even non-universal photonic processors can perform operations beyond the capabilities of classical devices^29,36–38^. The technological contribution of this work is to go a substantial step further and investigate to what extent the newly found levels of control and system size can be exploited for photonic quantum simulation of systems of interest, contributing to placing integrated optical devices in the realm of quantum technological devices^26,39–42^ for quantum simulation.

## Results

### Local equilibration

In any setting governed by closed-system Hamiltonian dynamics, equilibration can only happen locally for local observables since the global entropy must be preserved in time. In the setting considered, the global system is a multi-mode linear-optical system initially prepared in a highly non-Gaussian state *ρ* on *m* bosonic degrees of freedom, namely $\left|\psi \right\rangle \left\langle \psi \right|$ with $\left|\psi \right\rangle=\left|1,\ldots,\,1,\,0,\ldots,\,0\right\rangle$ of *n* = 3 single photons in *m* = 4 optical modes. The bosonic modes are associated with annihilation operators ${\hat{b}}_{1},\ldots,\,{\hat{b}}_{m}$. The subsequent integrated linear optical circuit is given by a unitary *V* ∈ U(*m*) that linearly transforms the bosonic modes. Any unitary from the group *U*(*m*) of *m* × *m* unitary matrices can be realized by a suitably designed linearly optical circuit. In state space, such linear optical circuits are reflected by *ρ* ↦ *σ* ≔ *U*(*V*)*ρU*(*V*)^†^, where *U*(*V*) is the physical implementation of the passive mode transformation *V* that linearly transforms a set of bosonic operators to a new set as ${({\hat{b}}_{1},\ldots,\,{\hat{b}}_{m})}^{T}\mapsto V{({\hat{b}}_{1},\ldots,\,{\hat{b}}_{m})}^{T}$. The representation of the mode transformation in Hilbert space *V* ↦ *U*(*V*) is commonly referred to as the metaplectic representation in technical terms. Finally, the output distribution is measured in the Fock basis using quasi-photon number resolving detectors, giving measurements of the form *μ* ↦ *P*(*μ*) with

1 $$P(\mu )=\langle {n}_{1},\ldots,\,{n}_{m}|U(V)\rho U{(V)}^{{{{\dagger}}} }|{n}_{1},\ldots,\,{n}_{m}\rangle,$$

where *μ* = (*n*_1_, …, *n*_*m*_) is a given pattern of detection events.

For our purpose of showing local equilibration, we interpret the evolution $U(V)={e}^{-i\hat{Ht}}$ as the evolution under a Hamiltonian $\hat{H}$ for time *t* > 0, which distributes information. In the linear optical system at hand, we will implement two Hamiltonians, a quadratic bosonic translationally invariant ‘hopping’ Hamiltonian, resembling the non-interacting limit of a Bose-Hubbard Hamiltonian, and a Haar random transformation *V* ∈ *U*(*m*) corresponding to a Hamiltonian with random long-range interactions. In a fixed-size optical system, we can simulate the evolution at various times by tuning the strength of the evolution, interpreting *t* as scaling the strength rather than the duration of the interaction.

As the time *t* gets larger, increasingly longer-ranged entanglement builds up. This means that the expected moments of the local photon number ${\hat{n}}_{j}:={\hat{b}}_{j}^{{{{\dagger}}} }{\hat{b}}_{j}$ of each of the output modes labeled *j* = 1, …, *m* of the state *σ* will increasingly, in the depth of the circuit, equilibrate and lead to a distribution that resembles that of a (generalized) Gibbs ensemble. In other words, as seen in Fig. 1, one encounters local equilibration where the reduced quantum states of a subset of the modes, or individual modes, equilibrate and take thermal-like values. Equivalently, we can say that the state will locally thermalize in the sense that it results in the same expectation values for local observables as if the entire system had relaxed to a thermal equilibrium state.

Strictly speaking, here we observe a generalized thermalization in the following sense. The Gibbs or canonical state reflecting thermal equilibrium is given by $\xi :={e}^{-\beta \hat{H}}/{{{{{{{\rm{tr}}}}}}}}({e}^{-\beta \hat{H}})$ for a suitable inverse temperature *β* > 0 that is set by the energy density. For non-interacting bosonic systems, local equilibration for subsystems consisting of several modes is instead expected to converge to a generalized Gibbs state. To be specific, here, the initial state is a product state (and hence has obviously short-ranged correlations)—albeit not being translationally invariant—and the bosonic quadratic Hamiltonian will, on the one hand, be translationally invariant before it undergoes a time evolution generated by $U(V)={e}^{-i\hat{Ht}}$ (or the Haar-random *V* ∈ *U*(*m*)). The situation is particularly transparent where $\hat{H}$ is a hopping Hamiltonian, which is translationally invariant. Defining the momentum space occupation numbers as

2 $${\hat{N}}_{k}:=\frac{1}{m}\mathop{\sum }\limits_{x,y=1}^{m}{e}^{2\pi ik(y-x)/m}{\hat{b}}_{x}^{{{{\dagger}}} }{\hat{b}}_{y}$$

one finds that the GGE is then given by the maximum-entropy state *ω* given by

3 $$\omega :=\,{{\mbox{argmax}}}\,\{S(\eta ):\,{{\mbox{tr}}}\,(\eta {\hat{N}}_{k})=\langle \psi|{\hat{N}}_{k}|\psi \rangle \,{{\mbox{for all}}}\,\,k\},$$

associated with an inverse temperature per momentum mode, where $S(\eta )=-{{{{{{{\rm{tr}}}}}}}}(\eta \log \eta )$ is the von Neumann entropy. For an infinite system, convergence to such a state is guaranteed^7–9,11^, in the sense that the global pure state will remain pure, but again, all reduced states (and, for that matter, all expectation values of local observables) will for most times take the values of this GGE. For finite systems, it has been rigorously settled in what sense the state is locally approximated by such a GGE^7,12–15^ before recurrences set in. We discuss the specifics of this mechanism in more detail in Supplementary Note 2. For the Haar-random unitaries, we still find Gaussification in expectation, creating an interesting state of affairs, as here, the theoretical underpinning is less clear.

For subsystems consisting of a single bosonic mode only, canonical or Gibbs states, as well as GGEs, both give rise to identical photon number distributions reflecting Gaussian states: The state ‘Gaussifies’ in time. The situation at hand is particularly simple in the situation where the expectation value of the photon number is the same for each of the *m* output modes. Then for a Gaussian state, the probability of observing *k* photons reduces to

4 $$p(k)=\frac{\left({{n-k+m-2}\atop{n-k}}\right)}{\left({{n+m-1}\atop{n}}\right)}=\frac{{D}^{k}}{{(D+1)}^{k+1}}\left\{1+O\left(\frac{1}{m}\right)\right\},$$

where *D* ≔ *n*/*m* is the photon density per mode, which acts as an effective temperature.

Interestingly, GGEs are still not quite thermal or canonical Gibbs states, which would be maximum-entropy states given the expectation value of the energy, but a generalization of that state, due to the non-interacting nature of the Hamiltonian. For example, in full non-equilibrium dynamics under large-scale interacting Bose-Hubbard Hamiltonians (as can be probed with cold atoms in optical lattices^21^), one expects an apparent relaxation to a Gibbs state. In contrast, a GGE maximizes the von Neumann entropy under the constraint of the energy expectation and the momentum space occupation numbers, which are preserved under the non-interacting translationally invariant evolution $t\,\mapsto \,{e}^{-i\hat{Ht}}$. Therefore, one can say that each of the momentum modes is then associated with its own temperature, as sketched in Fig. 1, and the system ‘thermalizes’ up to the constraints of the momentum space occupation numbers being preserved.

Such GGEs are also interesting from the perspective of quantum thermodynamics^19,43,44^. The presence of the additional conserved charges indeed alters the thermodynamic properties and comes in as a further constraint. It is also found that the minimum-work principle can break down in the presence of a large number of conserved quantities^43^. Resource theories for thermodynamic exchanges of non-commuting and hence non-Abelian observables are also strongly altered for GGEs compared to their thermal counterparts^44^.

### Certification

In this section, we lay out the certification tools that we have developed to verify that the experiment has worked close to its anticipated functioning. Crucially, time evolution preserves the purity of a quantum system; the system only appears to be equilibrated when considering the local dynamics. Therefore, in the ideal case, it should be possible to undo the time evolution after applying *U*. This leads to the evolution *U*^†^*U* = *I*, meaning that a revival of the initial, non-Gaussian state is observed. In a noiseless experiment, this operation would function perfectly, and all entanglement will be formed between the photons as opposed to between the photons and the environment. This latter form of entanglement corresponds to decoherence and cannot be time-reversed by acting only on the photons. Therefore, the extent to which one observes a revival of the initial state serves as a measure of the degree of photon-photon entanglement versus the degree of decoherence.

We further formalize this idea in the form of a fidelity witness^45,46^ that certifies the fidelity $F(\sigma,\,\left|{\psi }_{t}\right\rangle )=|\left\langle {\psi }_{t}\right|\sigma \left|{\psi }_{t}\right\rangle|$ between the experimentally prepared state *σ* and a pure target state described by a state vector $\left|{\psi }_{t}\right\rangle :={e}^{-i\hat{Ht}}\left|\psi \right\rangle$. The procedure requires a well-calibrated, programmable measurement unitary and number-resolving (but not spectral-mode resolving) detectors. It consists of two settings for the measurement unitary: the inverse of the target unitary and the inverse followed by a Fourier transform *U*_F_. The constant number of measurement settings and polynomial classical computation resources required means the procedure is efficiently scalable to arbitrary system sizes. Here, we consider the specific case of witnessing against the specific target state of our experiment, leaving the generalization to Supplementary Note 1.

For the first measurement, we measure the state *U*^†^*σU* in the photon number basis. More specifically, we measure the fraction *p*_1_ of detection events that correspond to our input state (i.e., exactly one photon in the first three input modes and no photon in the fourth mode). If our photodetectors would perfectly resolve the temporal and spectral degrees of freedom of the photons, this measurement in itself would be sufficient for certification^45^. However, in our system, the detectors only resolve the spatial mode. Neglecting this and naively carrying out the above procedure could result in certifying a large fidelity even with photons in distinct temporal modes, i.e., distinguishable states.

To rule this out, we employ an additional measurement setting as part of a two-step certification process: we implement *U*^†^ followed by a Fourier transformation and count photons. From the first setting, we upper bound the probability *p*_1_ of seeing one photon in each of the first three spatial modes and no photon in the fourth. From the second, we upper bound *p*_2_, the overlap probability of *σ* with the distinguishable sub-space. This is done by monitoring the fraction of observed interference patterns that would be forbidden for truly indistinguishable photons following a Fourier transform^47–49^.

In this way, we arrive at a fidelity bound of the form

5 $$F\ge {p}_{1}-\frac{9}{4}{p}_{2}-\delta (\epsilon )$$

where *ϵ* > 0 is the probability that the bound is correct, and *δ* is the corresponding statistical penalty, which arises from the observed photon counting statistics on *p*_1_ and *p*_2_. This bound is derived from Chebyshev’s inequality and holds with very few assumptions on the underlying distribution (for a full derivation, see Supplementary Note 1).

If one is merely interested in establishing the presence of entanglement in the system, one can derive a simple entanglement witness ${{{{{{{\mathcal{W}}}}}}}}$ from the estimated fidelity. We use the following definition of an entanglement witness^50^

6 $${{{{{{{\mathcal{W}}}}}}}}:={\lambda }_{\max }^{2}{\mathbb{I}}-\left|{\psi }_{t}\right\rangle \left\langle {\psi }_{t}\right|$$

where ${\lambda }_{\max }^{2}$ is the maximal Schmidt coefficient in the decomposition of $\left|{\psi }_{t}\right\rangle$ over a given partition, whose classical computation is not scalable but feasible in our case. It follows then that $F\, > \,{\lambda }_{\max }^{2}$ is a witness of entanglement.

### Integrated photonic platform

We use an integrated quantum photonics architecture as our experimental platform (see Fig. 2). Integrated quantum photonics constitutes a platform for non-universal quantum simulation based on bosonic interaction between indistinguishable photons^27,51–55^. In integrated quantum photonics, quantum states of light are fed into a large-scale tunable interferometer and measured by single-photon-sensitive detectors.

Our interferometer is realized in silicon nitride waveguides^56,57^, it has an overall size of *n* = 12 modes and an optical transmission of 2.2–2.7 dB, i.e., 54–60% depending on the input channel. Reconfigurability of the interferometer is achieved by a suitable arrangement of unit cells consisting of pairwise mode interactions realized as tuneable Mach-Zehnder interferometers^58^. Each unit cell of the interferometer is tuneable by the thermo-optic effect. For a full 12-mode transformation, the average amplitude fidelity $F={n}^{-1}{{{{{{{\rm{Tr}}}}}}}}(|{U}_{{{{{{{{\rm{set}}}}}}}}}^{{{{\dagger}}} }||{U}_{{{{{{{{\rm{get}}}}}}}}}|)$ is *F* = 0.98, where *U*_set_ and *U*_get_ are the intended and achieved unitary transformations in the processor, respectively. The processor preserves the second-order coherence of the photons^57^.

We implement a quantum simulation of thermalization and a verification experiment in two separate sections of the interferometer. These two sections are indicated in blue and yellow, respectively, in Fig. 2; the area below the dotted line in Fig. 2 is not used. These two sections both form individual universal interferometers on the restricted space of four optical modes, allowing us to apply two arbitrary optical transformations *U*_1_ and *U*_2_, in sequence.

We use the first section to simulate the time evolution of our input state. We select two families of Hamiltonians to simulate: A hopping Hamiltonian ${\hat{H}}_{{{{{{{{\rm{NN}}}}}}}}}=\gamma {\sum }_{k}{\hat{b}}_{k}^{{{{\dagger}}} }{\hat{b}}_{k+1}+{{{{{{{\rm{h.c.}}}}}}}}$ which consists of equal-strength nearest-neighbor interactions between all modes, which simulates the superfluid, non-interacting limit of the Bose-Hubbard model, and a set of 20 randomly chosen long-range Hamiltonians ${\hat{H}}_{{{{{{{{\rm{LR}}}}}}}}}={\sum }_{i,j}{\gamma }_{i,j}{\hat{b}}_{j}^{{{{\dagger}}} }{\hat{b}}_{i}+{{{{{{{\rm{h.}}}}}}}}\,{{{{{{{\rm{c.}}}}}}}}$, which we generate by applying the matrix logarithm to a set of Haar-random unitary matrices^59^.

The second section of the interferometer, indicated in Fig. 2 in yellow, is used for certification. When we wish to directly measure the quantum state generated by the first section, we set this area to the identity, leaving the state after *U*_1_ untouched. However, we can also use this second section to make measurements in an arbitrary basis on the quantum state generated by *U*_1_, which allows us to certify the closeness of our produced quantum state to the ideal case.

Our photon source is a pair of periodically poled potassium titanyl phosphate (ppKTP) crystals operated in a Type-II degenerate configuration, converting light from 775 to 1550 nm^60^, with an output bandwidth of Δ*λ* ≈ 20 nm. By using a single external herald detector and conditioning on the detection of three photons after the chip, we post-select on the state vector $\left|\psi \right\rangle=\left|1,\,1,\,1,\,0\right\rangle$^52^. By tuning the relative arrival times of our photons, we can continuously tune the degree of distinguishability between our photons. On-chip measurements via the Hong-Ou-Mandel (HOM) effect^61^ lower bound the wave function overlap between photons *x* = ∣〈*ψ*_*i*_∣*ψ*_*j*_〉∣, according to *V* = *x*^2^, where $\left|{\psi }_{i}\right\rangle$ is the wave function of photon *i*, and *V* is the visibility of the HOM dip. We measure the visibility of 89% and 92% for photons of different sources and 94% for photons of the same source. Photon detection is achieved with a bank of 13 superconducting single-photon detectors^62,63^, which are read out with standard correlation electronics. For each of our four modes of interest, we multiplexed three detectors to achieve quasi-photon number resolution^64^, with the thirteenth detector used as the herald. By means of adjusting the time delay between the photons, we can adjust their degree of mutual distinguishability. We can switch between indistinguishable particles, which produce an overall entangled state (i.e., exhibiting both modal and particle entanglement), which will exhibit thermalization, and distinguishable particles, in which each photon traverses the experiment unaffected by the others, corresponding to a product state of the single-photon wave functions, which does not exhibit local thermalization.

### Experimental results

**Fig. 3:**
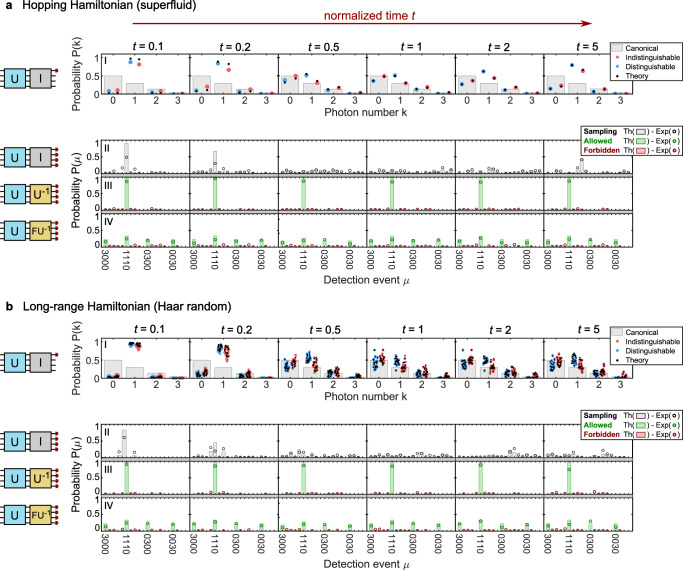
Quantum simulation of single- and multi-mode measurements. **a** Hopping Hamiltonian (superfluid): In panel I, the time evolution of photon-number probability distribution in spatial output mode 1 is plotted. The black points (squares) show the theoretical prediction for indistinguishable (distinguishable) particles, while colored points correspond to experimental data. Panels II–IV show the observed output distributions. These rows correspond to the output distributions of the hopping Hamiltonian (panel II), the first certification measurement *U*^−1^ (panel III), and the second certification measurement *U*_*F*_*U*^−1^ (panel IV). Theoretical predictions (Th) are represented by bars, and the experimental results (Exp) are represented by circles. The green-colored data corresponds with outcomes that benefit the certification protocol, whereas the red data is forbidden, i.e., ideally, should not occur. **b** Long-range Hamiltonian (Haar random): In panel I, the time evolution of photon-number probability distribution in spatial output mode 1 for 20 different random Hamiltonians is plotted. The black points (squares) show the theoretical prediction for indistinguishable (distinguishable) particles, while colored points correspond to experimental data. Panels II–IV show the observed output distributions for the first long-range Hamiltonian. These rows correspond to the output distributions of the first long-range Hamiltonian (panel II), the first certification measurement *U*^−1^ (panel III), and the second certification measurement *U*_*F*_*U*^−1^ (panel IV). Theoretical predictions (Th) are represented by bars, and the experimental results (Exp) are represented by circles. The green-colored data corresponds with outcomes that benefit the certification protocol, whereas the red data is forbidden, i.e., ideally, should not occur.

Figure 3 shows the results of our quantum simulation of the hopping Hamiltonian and 20 random instances of longe-range Hamiltonians in sub-figures (a) and (b), respectively. The two sub-figures each have a tabular structure, where the columns indicate the different simulated time steps, with the simulation time indicated at the head of the column, and the rows indicate different measurement settings, i.e., either the experiment itself or the corresponding certification measurements. The data in these figures were acquired over 20 min for the photon number distribution, 320 min per certification measurement for the hopping Hamiltonian, and 220 min for each certification measurement of the long-range Hamiltonian, with four-photon events (three photos in the processor plus herald) occurring at a rate of 4 Hz.

The first row of the two sub-figures displays the single-mode photon-number statistics *k* ↦ *p*(*k*) as generated after the application of *U* in the first section of the processor. The output statistics were measured for the first output mode. The experiment was carried out for both distinguishable (blue points) and indistinguishable (red points) photons. The gray bars show the expected distribution at full equilibration given by Eq. (4). For both Hamiltonians, initially, the input state is still clearly present, as indicated by the high probability of observing exactly one photon in the observed output mode. However, entanglement builds up as time evolves since the photons increasingly equilibrate. Consequently, for the indistinguishable photons, the initial input state evolves to a thermal-like state at *t* = 1. For both Hamiltonians, the distinguishable photons (whose output statistics correspond to those of classical particles) do not approach the canonical thermal state, demonstrating the intrinsic link between entanglement and thermalization.

For the hopping Hamiltonian, at later times (*t* = 2, *t* = 5), the finite size of our Hamiltonian gives rise to a *recurrence*, i.e., the state moves away from equilibrium again and evolves back towards the initial input state^7,8^. For the long-range Hamiltonian, in contrast, the long-range interactions mean that recurrences are pushed away to a later time not included in the simulation. These results suggest the presence of long-range order (as opposed to structured, nearest-neighbor interactions) tends to increase the time for which a system will continue to exhibit local relaxation. While this picture is intuitive, a rigorous understanding of these effects is an exciting open problem for theory and future experiments. The general agreement across a large range of randomly chosen Hamiltonians also represents strong experimental evidence for the ubiquity of these effects^5,6^.

The second row of the two sub-figures shows the full output-state distribution *μ* ↦ *p*(*μ*) after only the application of *U*, measured with indistinguishable photons. The bars in the background correspond to the expected distributions. For the long-range Hamiltonian, a single representative example of our 20 Hamiltonians is plotted. From this data, it can be clearly seen that at the point of thermalization, the photons are spread over many possible output configurations, whereas a recurrence manifests as a transition back to fewer possible output configurations.

The third and fourth rows show the output-state distributions after the first and second certification measurement, respectively. In these rows, the output configurations which contribute positively to the fidelity witness are indicated in green, and those which contribute negatively are indicated in red. The first certification measurement undoes the entanglement generated by *U* and ideally only results in state vectors of the form $\left|{\psi }_{{{{{{{{\rm{out}}}}}}}}}\right\rangle=\left|1,\,1,\,1,\,0\right\rangle$. The second certification measurement also applies a three-mode Fourier to the generated states. Ideally, this results in only four allowed output configurations. These certification measurements show good agreement with the ideal allowed states, demonstrating a high degree of control over the experiment. For the second certification measurement, most of the deviations from the expected distribution can be attributed to the known photon indistinguishability. From the data presented in the third and fourth row, we extract the values of *p*_1_ and *p*_2_, respectively, which are used in the fidelity witness as laid out in Eq. (5).

**Fig. 4:**
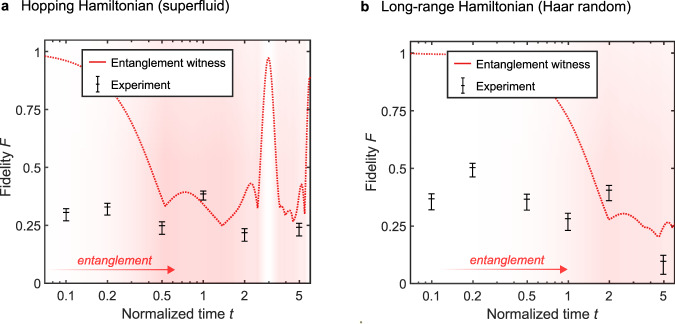
Global state fidelity certification. **a** Certification of entanglement in the hopping Hamiltonian (superfluid): The lower bound certification fidelity estimations for the hopping Hamiltonian are plotted against a theoretical entanglement witness. **b** Certification of entanglement in the first long-range Hamiltonian (Haar random): The lower bound certification fidelity estimations for the first long-range Hamiltonian are plotted against a theoretical entanglement witness. In both plots, the top, middle, and bottom points at each time step correspond to confidence values of *ϵ* = 0.7, *ϵ* = 0.8, and *ϵ* = 0.9, respectively. The background color saturation qualitatively shows the total entanglement generated at that time step, which is proportional to the value of the entanglement witness. A higher saturation indicates a stronger presence of multi-photon entanglement.

Figure 4a, b shows the certified fidelities for both the hopping Hamiltonian and the first random long-range Hamiltonian, respectively. The three horizontal ticks on each data point correspond to confidence values of *ϵ* = 0.7, *ϵ* = 0.8, and *ϵ* = 0.9. The line shows the entanglement witness, corresponding to a bi-partition between mode 1 and the remaining modes. The relatively constant fidelity to the target global state contrasts against the conversion of the local, single-mode statistics to thermal statistics, as seen in Fig. 3.

Figure 4 a) shows that entanglement is certified for *t* = 1 in the hopping Hamiltonian system. The observed fidelity *F* = 0.359 is above the threshold of the entanglement witness. Similarly, Fig. 4b) shows an unambiguous certification for the first long-range Hamiltonian at *t* = 2. The fidelity *F* = 0.360 is well above the certification threshold. Both of these entanglement certifications hold with a confidence of at least 90%.

The certification fidelities are limited by imperfect control over the processor. This follows from the certification fidelity at *t* = 0.2 for the long-range Hamiltonian. This fidelity *F* = 0.462 is significantly higher than others. Closer inspection shows a near-optimal value for *p*_2_, which is now only limited by the partial distinguishability of the generated photons. This implies that the certification at other time steps is limited by imperfect chip control, i.e., a limited fidelity at which any measurement can be implemented. A second factor limiting the certification is detector blinding, which affects the obtained values of *p*_1_ (see Supplementary Notes 5 and 6 for more details on detector blinding and the convergence of the certification statistics).

## Discussion

In conclusion, we have experimentally shown that a pure quantum state in a closed environment can locally behave like a thermal state because of entanglement with the other modes. To this end, we simulated both the non-interacting limit of a Bose-Hubbard hopping Hamiltonian and 20 random long-range Hamiltonians on a programmable 12-mode photonic processor. Previous experiments in this direction have not been able to show this kind of reversibility since creating a sufficiently isolated quantum system and controllable evolution is notoriously difficult. However, our experiment is fully time-reversible, just like quantum mechanics itself. This reversibility has allowed us to certify that equilibration and thermalization are due to entanglement between the quantum particles rather than with the environment. These results also provide experimental evidence for the universality of these phenomena and shed new light on the role of long-range interactions on relaxation dynamics. From the point of view of the development of quantum technologies, these experiments showcase the degree of control, low decoherence, and rapidly growing size of integrated quantum photonic processors as instances of a near-term quantum computational platform.

## Methods

### Photon source and input state preparation

Distinguishable and indistinguishable photonic quantum state vectors of the form $\left|\psi \right\rangle=\left|1,\,1,\,1,\,0\right\rangle$ are generated by a multi-photon source consisting of two free-space Type-II SPDC sources. Two non-linear 2 mm length ppKTP crystals (Raicol Crystals) are pumped by a Ti:Sa mode-locked laser (Tsunami, Spectra-Physics) at 775 nm with a spectral bandwidth of 5.4 nm FWHM. Pulses are generated with a repetition frequency of 80 MHz and 150 fs pulse duration. Each crystal is pumped by approximately 10 mW pump power, generating degenerate signal-idler pairs at 1550 nm with a generation probability <1% per pulse. Typical heralding efficiencies for individual crystals are around 40–45%, while typical two-photon event rates are ~0.20 MHz coincidence counts at 40 mW pump power. While the source is designed to produce as pure photons as possible, residual energy and momentum conservation result in spectral signal-idler correlations. These correlations are attributable to the periodically poled structure of the non-linear crystals. We suppress these correlations by using a spectral bandpass filter of Δ*λ* = 25 nm. Halfwave plates are used to remove the distinguishability in photon polarization and to match the TE mode supported by our quantum photonic processor. Three motorized linear stages (SLC-2475, Smaract GmbH) are used to control relative photon arrival times used to switch the distinguishability of the photons.

### Quantum photonic processor

Our quantum photonic processor consists of a photonic chip, the control electronics which actuate this chip, and peripheral systems such as cooling. The photonic chip implements arbitrary linear optical transformations on 12 waveguides. The waveguides are implemented as stoichiometric silicon nitride (${{{{{{{{\rm{S{i}}}}}}}_{3}{{{{{\rm{N}}}}}}}}}_{{{{{{{{\rm{4}}}}}}}}}$) asymmetric double-stripe (ADS) waveguides with the TriPleX technology^56^. The waveguides are optimized for light of a wavelength of 1550 nm and have propagation loss of <0.1 dB/cm. The waveguides have a minimum bending radius of 100 µm. Coupling on and off the chip is achieved by adiabatic mode converters, which are implemented by removing the top layers of the ADS stack. These converters have coupling losses down to 0.9 dB/facet. The overall measured loss budget of the processor is 2.5 ± 0.2 dB, with roughly 1.8 dB attributable to the two adiabatic couplers and 0.7 dB to propagation losses on the chip.

The universality of the optical transformation is achieved by a network of beam splitters in a checkerboard geometry. Each tunable beam splitter is implemented as a Mach-Zehnder interferometer (MZI) with two static 50/50 directional couplers. To tune the MZI, two thermo-optical phase shifters are used, one inside the MZI, which enables shifting of light amplitude between adjacent optical modes, and one external to the MZI, which allows for a relative phase shift between the two modes. The thermo-optic phase shifters are implemented as 1 mm long platinum heaters, have *V*_*π*_ = 10 V, and dissipate roughly 400 mW of power each. This power is carried off the chip through a Peltier element which is itself actively cooled with water cooling. A bank of 132 digital-to-analog converters converts signals from a control computer to voltages over the heaters. A dedicated software package is used for communication and to compute the required voltages. Control over the processor to the precision required in this experiment requires an understanding of the crosstalk between these control channels, which is achieved in a dedicated software package.

### Photon detection system

A suite of 13 superconducting nanowire single-photon detectors (SNSPDs) is used for photon detection. These detectors are biased close to their critical current (8–22 μA range), operating at quantum efficiencies of around 90% for 1550 nm photons with typical 200 Hz dark counts. Fourfold coincidence rates within a 750 ps window are monitored by a time tagger device (Timetagger Ultra, Swabian). From the combination of photon generation rates and dark count rates, we estimate that less than one in a million measured four-fold coincidence events are expected to be triggered by a dark count. Polarization-maintaining fibers are used in combination with polarization controllers to optimize and stabilize output counts in each channel. Pseudo-number resolution detection is realized by multiplexing detectors in a 1-to-3 quasi-photon number resolving detector (q3PNRD) configuration by fiber-beam-splitters on the four optical modes of interest, with the thirteenth detector used as a herald.

### Photon detection calibration

In order to sample from *μ* ↦ *P*(*μ*) in an unbiased way, as required in this work, it is important to characterize the relative output losses from the different detectors. The SNSPDs have variations in their detection efficiency, and the same holds for the output coupling of the various optical modes of the photonic processor. Non-uniformity in the overall detection efficiency of our experiment biases the sampling of *P*(*μ*) since it will suppress some outcomes while relatively enhancing others. Note that this does not hold for any inhomogeneities in the in-coupling due to post-selection. Furthermore, we assume that on-chip losses are reasonably uniform, which is evidenced by the high matrix amplitude fidelities. Furthermore, note that an absolute detection calibration (a notoriously difficult problem at the single-photon level) is not necessary, only a relative one between the 12 detectors of interest.

Non-uniform detection channel losses are characterized by directly transmitting heralded single photons from input mode 1 to all four output modes consecutively; these optical transformations can be performed with high fidelity. In each of these four consecutive experiments, the heralded singles count rate of each detector in the q3PNRD behind the output mode of interest is measured. All measured heralded singles count rates *S*_*i*_ originate from the same on-chip uniform heralded single-photon rate *R*_1_. Therefore, it is convenient to pool all other losses such as out-coupling efficiencies, detection efficiencies, and splitting ratios for each detection channel *i* in a lumped factor *p*_*i*_, to get

7 $${S}_{i}={p}_{i}{R}_{1}.$$

Since we are only interested in relative efficiencies, we introduce relative weight factors for each detection channel, which are then normalized with respect to the maximum measured heralded singles rate and defined by

8 $${w}_{i}=\frac{{S}_{i}}{{S}_{\max }}.$$

In our experiments, we achieved excellent weight factor stability. Typically, we observed less than 1% relative fluctuations over more than 15 h time span.

Similar to nonuniform detection efficiency, the fact that each q3PNRD is effectively less efficient when detecting multiple photons as opposed to a single photon biases the output distribution and must be corrected. Experimentally, we measure heralded threefold coincidence rates *CC*_*p*,*q*,*r*_, which denote the rate at which detectors *p*–*r* and the herald detector fire simultaneously, normalized to the overall frequency of successful experiments. The challenge is then to convert these probabilities into an unbiased estimate of *P*(*μ*).

To compensate for q3PNRD effects, we enumerate all combinations of threefold detection events which would give rise to a particular output pattern *μ*. For probabilistic multi-photon detection, the probability of measuring *j* photons behind mode *i* when *k* photons are injected is denoted *P*_*i*_( *j*∣*k*). We note that for *P*_*i*_(1∣1) and *P*_*i*_(2∣2), there are three possible permutations, while for *P*_*i*_(3∣3), there is just one permutation. More explicitly, we find

9 $${P}_{i}(0|0)=1,$$

10 $${P}_{i}(1|1)={w}_{{p}_{i}}+{w}_{{q}_{i}}+{w}_{{r}_{i}},$$

11 $${P}_{i}(2|2)=2!({w}_{{p}_{i}}{w}_{{q}_{i}}+{w}_{{q}_{i}}{w}_{{r}_{i}}+{w}_{{p}_{i}}{w}_{{r}_{i}}),$$

12 $${P}_{i}(3|3)=3!{w}_{{p}_{i}}{w}_{{q}_{i}}{w}_{{r}_{i}},$$

where *w* are the weight factors determined above and ${w}_{{p}_{i}}+{w}_{{q}_{i}}+{w}_{{r}_{i}}\le 1$ due to incorporated losses. Since all *P*_*i*_(*j*∣*k*) are independent probability events, we find an estimate for *P*(*μ*)

13 $$P(\mu )=\frac{{\sum }_{(p,q,r)\in \mu }C{C}_{p,q,r}}{{P}_{1}({n}_{1}|{n}_{1}){P}_{2}({n}_{2}|{n}_{2}){P}_{3}({n}_{3}|{n}_{3}){P}_{4}({n}_{4}|{n}_{4})},$$

where *μ* = (*p*, *q*, *r*) denotes all combinations of detection events contributing to the same *μ* and *n*_*i*_ is the number of photons detected in a mode *i* for a given *μ*. These results are used to correct raw measurement data.

## Supplementary information

Supplementary Information

## Data Availability

All experimental and simulated data used in this study are available in the 4TU.ResearchData database^65^.

## Code availability

All data post-processing and simulation codes used in this study are available in the 4TU.ResearchData database^65^.
