# Supplementary material for: Quantum simulation of thermodynamics in an integrated quantum photonic processor
Source: Nat Commun. 2023 Jul 1;14:3895. doi: 10.1038/s41467-023-38413-9 (PMC10314952; doi:10.1038/s41467-023-38413-9)
Supplement: Supplementary file 1 — Supplementary Information [file 41467_2023_38413_MOESM1_ESM.pdf]

Supplementary Information underlying the manuscript: "Quantum simulation of thermodynamics in an integrated quantum photonic processor"

F. H. B. Somhorst,[1] R. van der Meer,[1] M. Correa Anguita,[1] R. Schadow,[2] H. J. Snijders,[3] M. de Goede,[3] B. Kassenberg,[3] P. Venderbosch,[3] C. Taballione,[3] J. P. Epping,[3] H. H. van den Vlekkert,[3] J. Timmerhuis,[1] J. F. F. Bulmer,[4] J. Lugani,[5] I. A. Walmsley,[6,7] P. W. H. Pinkse,[1] J. Eisert,[2,8,9] N. Walk,[2] and J. J. Renema[1,3]

[1]*MESA+ Institute for Nanotechnology, University of Twente, P. O. box 217, 7500 AE Enschede, The Netherlands*
[2]*Dahlem Center for Complex Quantum Systems, Freie Universität Berlin, 14195 Berlin, Germany*
[3]*QuiX Quantum B.V., Hengelosestraat 500, 7521 AN Enschede, the Netherlands*
[4]*Quantum Engineering Technology Labs, University of Bristol, Bristol, United Kingdom*
[5]*Center for Sensors, Instrumentation and Cyber Physical System Engineering, IIT Delhi, New Delhi 110 016, India*
[6]*Department of Physics, Imperial College London, Prince Consort Rd., London SW7 2AZ, United Kingdom*
[7]*Clarendon Laboratory, University of Oxford, Parks Road, Oxford OX1 3PU, United Kingdom*
[8]*Helmholtz-Zentrum Berlin für Materialien und Energie, 14109 Berlin, Germany*
[9]*Fraunhofer Heinrich Hertz Institute, 10587 Berlin, Germany*

SUPPLEMENTARY NOTE 1 – DERIVATION OF FIDELITY WITNESS

A fidelity witness provides guarantee that the fidelity of some target state with an experimental output is at least a certain threshold value with at least a certain probability [1]. Here, we present a derivation of such a witness, including finite-size statistics, that is efficient in terms of experimental effort and classical computation. In the first place we can use this threshold as evidence that our global system retains approximately the same fidelity with a target pure state whilst the local systems exhibit apparent entropy increase. A natural further question that arises is, is a particularly meaningful fidelity threshold? In this experiment, where the key feature of interest is the role of entanglement in producing local entropy production, we will use previously established relationships between fidelity and entanglement (see, e.g., Supplementary Ref. [2] to establish useful benchmarks). The idea is that the fidelity between a separable state and an entangled target state vector $|\psi_t\rangle$ cannot exceed a certain threshold, which is set by the largest Schmidt coefficient. If the fidelity exceeds that threshold, i.e., if $F > \lambda_{\max}^2$, entanglement must be present. Because the size of the largest Schmidt coefficient decreases with the amount of entanglement, for more entangled states lower fidelities are sufficient to witness the presence of entanglement.

Ideal case: Fully-mode-resolving detectors. The fidelity between a quantum state σ and a target state $\sigma_t = |\psi_t\rangle\langle\psi_t|$ is defined as

$$F(\sigma, \sigma_t) := \mathrm{tr}(\sigma\sigma_t) = \langle\psi_t|\,\sigma\,|\psi_t\rangle\,. \tag{1}$$

In our case, the target state vector is an initial state vector

$$|\psi\rangle = |\underbrace{1,\ldots,1}_{n},\underbrace{0,\ldots,0}_{m}\rangle \tag{2}$$

of single photons in the first n modes of an m mode system, evolved by a unitary, $U(V)$, implementing a passive linear optical transformation V i.e., $\sigma_t = |\psi_t\rangle\langle\psi_t| = U(V)\,|\psi\rangle)\,\langle\psi|\,U(V)^\dagger$. Thus, the fidelity can then be written as

$$F = F(\sigma, U\,|\psi\rangle\langle\psi|\,U^\dagger) = F(U^\dagger\sigma U, |\psi\rangle\langle\psi|) \tag{3}$$

where we have suppressed the argument V for brevity, and is lower bounded in terms of photon number operators by [1]

$$F^{(n)} = \left\langle (n+1-\hat{n})\prod_{j=1}^{n}\hat{n}_j \right\rangle_{U^\dagger\sigma U}, \tag{4}$$

where $\hat{n}_j = \sum_{n_j=0}^{\infty} n_j\,|n_j\rangle\langle n_j| = \hat{b}_j^\dagger\hat{b}_j$ are the photon number operators, whose eigenvalues are the number of photons in mode j and $\langle\sum_j \hat{n}_j\rangle = n$ is the global photon number. When one post-selects for a constant global photon number in each run (which we do in our experimental setup, to $n = 3$), then the bound simplifies to

$$F^{(n)} = \left\langle \prod_{j=1}^{3}\hat{n}_j \right\rangle_{U^\dagger\sigma U}. \tag{5}$$

Note that the only calculation necessary is to compute the Hermitian conjugate of the given matrix U.

Real case: Spatial-mode-resolving detectors. In our experimental setup we do not have fully-mode-resolving detectors, meaning that we have no physical equivalent of $\hat{n}_j$. In particular, our detectors can only resolve spatial modes and no temporal ones. This leaves an uncertainty regarding the exact mode of the photon after the measurement. Instead of projecting onto a unique mode (a single pure quantum state $M_k = |k\rangle\langle k|$), our detectors project onto a set of states, which are spread out over the temporal degrees of freedom and, without further work at least, cannot be distinguished. This uncertainty severely limits the fidelity that can be established. For three temporal modes, the measurements at each of the four spatial modes correspond to the following set of operators,

$$\begin{aligned} M_0 &= |0,0,0\rangle\langle 0,0,0|\,, \\ M_1 &= |1,0,0\rangle\langle 1,0,0| + |0,1,0\rangle\langle 0,1,0| + |0,0,1\rangle\langle 0,0,1|\,, \\ M_2 &= |2,0,0\rangle\langle 2,0,0| + |0,2,0\rangle\langle 0,2,0| + |0,0,2\rangle\langle 0,0,2| \\ &\quad + |1,0,1\rangle\langle 1,0,1| + |1,1,0\rangle\langle 1,1,0| + |0,1,1\rangle\langle 0,1,1|\,, \\ M_3 &= |3,0,0\rangle\langle 3,0,0| + |0,3,0\rangle\langle 0,3,0| + \ldots\,, \end{aligned} \tag{6}$$

where M_0 detects the absence of photons and M_1, M_2 and M_3 measure one, two and three photons, respectively, in a given spatial mode. Since we set up our experiment such that the ideal initial and the target state consists of one photon per spatial mode, our measurement operator of interest will be M_1 and our certification scheme will be based on the measurement of $M_1^A \otimes M_1^B \otimes M_1^C \otimes M_0^D$, where A, B, C and D label the four spatial modes. We will want to verify whether the initial state is being recovered after implementing the unitary and its inverse, i.e., that the three photons are in the same temporal mode and each in a different one of the first three spatial modes. The problem with using these measurements and naively applying the bound in Supplementary Eq. (5) is that, considering Supplementary Eq. (6), it is clear these measurements can produce the ideal click pattern even if the photons were completely distinguishable and no quantum interference or multi-photon entanglement has been present.

At this point we introduce some alternative notation that will come in handy later: The numbers in the ket-vector label the occupied temporal mode (there are three temporal modes, so the numbers go from 1 to 3) and the subscript 1 indicates that each spatial mode is occupied by one photon only (which is the case for M_1). For example, all photons being in the first temporal mode reads $|1,1,1\rangle_1 = |1,0,0;1,0,0;1,0,0;0,0,0\rangle$. The first photon in the first, second photon in the second, and third photon in the third temporal modes reads $|1,2,3\rangle_1 = |1,0,0;0,1,0;0,0,1;0,0,0\rangle$.

Now that we have pointed out the ambiguity of just counting photons in spatial modes, and equipped with useful notation, we next turn to the question of how to overcome the uncertainty regarding the temporal or spectral degree of freedom. An answer lies in the observation that certain interference patterns can be clearly associated with non-synchronous, i.e., distinguishable, photon states (similar to a HOM dip [3]) – we call those interference patterns *forbidden patterns*. We make use of this effect in practice by implementing a Fourier transform U_F after the unitary U and its inverse and use the overlap between the distinguishable subspace of states and the image of the forbidden patterns under the Fourier transform U_F to sharpen the lower bound on the fidelity. It is worth mentioning that this overlap is not 1:1 and some ambiguity will remain. It does, however, reduce the ambiguity significantly and thereby increases the estimated fidelity in a useful way.

We now give a full derivation of the fidelity bound. The bound, as in Main Eq. (5), has two components. 1) A lower bound p_1 on seeing one photon per spatial mode and 2) an upper bound p_2 on the overlap of σ with the distinguishable subspace. The two components correspond to two different measurement settings:

1. Implement the unitary and its inverse and count photons.
2. Implement the unitary and its inverse, implement a Fourier transform (an interference experiment) and then count photons.

First measurement setup. For simplicity and without loss of generality we fix M_1^A to the first temporal mode, i.e., $M_1^A = |1,0,0\rangle\langle 1,0,0|$. The first measurement setup can be expressed as the operator product $|1,0,0\rangle\langle 1,0,0| \otimes M_1^B \otimes M_1^C \otimes M_0^D$. The overlap of the state σ with $|1,0,0\rangle\langle 1,0,0| \otimes M_1^B \otimes M_1^C \otimes M_0^D$, i.e., the probability of seeing one photon in each of the first three spatial modes (regardless of the temporal modes), can be estimated experimentally with accuracy ϵ_1. This estimation takes the form of a lower bound p_1 – the result of the first round of measurements.

$$\mathrm{tr}[U^\dagger \sigma U(|1,0,0\rangle\langle 1,0,0| \otimes M_1^B \otimes M_1^C \otimes M_0^D)] \geq p_1 \tag{7}$$

which expanded yields

$$\begin{aligned} &\mathrm{tr}\big[U^\dagger \sigma U(\\ &|1,1,1\rangle_1\langle 1,1,1|_1 + |1,1,2\rangle_1\langle 1,1,2|_1 + |1,1,3\rangle_1\langle 1,1,3|_1 + \\ &|1,2,1\rangle_1\langle 1,2,1|_1 + |1,3,1\rangle_1\langle 1,3,1|_1 + |1,2,2\rangle_1\langle 1,2,2|_1 + \\ &|1,3,3\rangle_1\langle 1,3,3|_1 + |1,2,3\rangle_1\langle 1,2,3|_1 + |1,3,2\rangle_1\langle 1,3,2|_1)\big] \\ &\geq p_1. \end{aligned} \tag{8}$$

The first term (black) reflects the fidelity given by $F = \mathrm{tr}\big[U^\dagger \sigma U(|1,1,1\rangle_1\langle 1,1,1|_1)\big]$. The other terms correspond to the overlap of $U^\dagger \sigma U$ with those states where one (blue) or two (red) photons are distinguishable. We summarize those states as $\hat{P}_1$ and $\hat{P}_2$, respectively, and call $\hat{P} = \hat{P}_1 + \hat{P}_2$ the distinguishable subspace. With that shorthand notation Supplementary Eq. (8), simplifies to

$$F \geq p_1 - \mathrm{tr}\big(U^\dagger \sigma U \hat{P}\big). \tag{9}$$

We can calculate p_1 by counting the relative number of instances of M_1 in our first measurement setup.

Second measurement setup. Next, in order to improve the fidelity bound in Supplementary Eq. (9), we need to upper bound its second term $\mathrm{tr}(U^\dagger \sigma U \hat{P})$, which enters the fidelity bound with a minus sign. This term contains the overlap of σ with the distinguishable subspace $\hat{P}$. We upper bound it by implementing a Fourier interference experiment on the first three modes. This is described by the unitary $U_F = U_3^{\mathrm{Four}} \otimes \mathbb{I}$, where $U_n^{\mathrm{Four}}(V_n^{\mathrm{Four}})$ is the appropriate Hilbert space operator corresponding to the physical implementation of the mode transformation

$$(V_n^{\mathrm{Four}})_{j,k} = \frac{1}{\sqrt{n}} e^{i2\pi(j-1)(k-1)/n}. \tag{10}$$

Ideally, the first three modes should each be occupied by one perfectly indistinguishable photon (the first term in Supplementary Eq. (8)). For a state of this form, some counting patterns corresponding to projections onto certain output states are impossible [4–6]. By contrast, as we will now show, situations involving anything other than the ideal case *will* result in some forbidden measurement outcomes with a certain probability (Supplementary Fig. 1). Working backwards from this, we can use the observed frequency of the forbidden states to get a worst case upper bound on $\mathrm{tr}(U^\dagger \sigma U \hat{P})$. This kind of discrimination is something we could not do using only the first setup. In this case, the forbidden patterns of photon numbers are all those which correspond to state vectors not included in the set $\{|1,1,1,0\rangle, |3,0,0,0\rangle, |0,3,0,0\rangle, |0,0,3,0\rangle\}$ [4–6]. Defining the projection onto all forbidden states as M_f, the quantity obtained with our second measurement setup can be written as

$$\mathrm{tr}\big(U_F U^\dagger \sigma U U_F^{-1} M_f\big) = \mathrm{tr}\big(U^\dagger \sigma U U_F^{-1} M_f U_F\big) \leq p_2, \tag{11}$$

where p_2 is the the probability of observing a forbidden state. Using the cyclicity of the trace in the middle term we see we can also interpret p_2 as the overlap of $U^\dagger \sigma U$ with the image of the forbidden states based on the photon counting measurements.

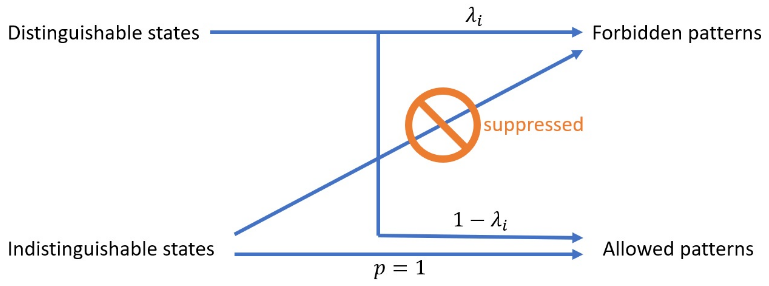

Supplementary Figure 1. **Forbidden measurement probability.** This figure shows how distinguishable and indistinguishable states translate to forbidden and allowed interference patterns after the Fourier transform. Forbidden patterns are strictly suppressed for indistinguishable states (in fact they are asserted via a suppression rule), hence indistinguishable states result in allowed patterns with unit probability. Distinguishable states result in forbidden patterns with probability λ_i, which we compute for all n-photon distinguishable states in all distinguishable sub-spaces P_i to establish a bound on $\mathrm{tr}(U^\dagger \sigma U \hat{P})$, the overlap between the state $U^\dagger \sigma U$ and the distinguishable subspace $\hat{P}$.

As mentioned before, some states within that image clearly correspond to distinguishable states, that is, states in $\hat{P}$ and other do not. To be more clear, we can express the image operator in terms of the distinguishable sub-spaces $P_i \in \hat{P}$ ($i \in \{1, 2\}$) as,

$$U_F^{-1} M_f U_F = \sum_i \lambda_i P_i + \hat{P}_\perp \tag{12}$$

where $\lambda_i := \mathrm{tr}(P_i U_F^{-1} M_f U_F)$ is the probability that a state in the distinguishable subspace $P_i \in \hat{P}$ results in a forbidden pattern and $\hat{P}_\perp$ projects onto the complementary space to $\hat{P}$. Since $\mathrm{tr}(U^\dagger \sigma U \hat{P}_\perp) \geq 0$, we have

$$\mathrm{tr}\big(U^\dagger \sigma U \sum_i \lambda_i P_i\big) \leq \mathrm{tr}\big(U^\dagger \sigma U U_F^{-1} M_f U_F\big) \leq p_2. \tag{13}$$

To calculate these probabilities what really matters is the number of mutually distinguishable photons. For example, to calculate the probability of the forbidden state vector $|2, 0, 1, 0\rangle$ from states in P_1 with one photon distinguishable from two other identical photons), one first computes the probability of the indistinguishable photons transforming through the Fourier transform into a state that could be transformed into a forbidden pattern by the third photon. This can be computed via Supplementary Eq. (24) derived in Supplementary Ref. [5]. In this case, that would be permutations of the outputs $|1, 0, 1, 0\rangle$ or $|2, 0, 0, 0\rangle$. Then one computes the probability that the final, distinguishable photon, would fall in the correct mode to produce the forbidden output. For example, if the indistinguishable photons evolve the state vector $|1, 0, 1, 0\rangle$, then the distinguishable boson evolving to either $|1, 0, 0, 0\rangle$ or $|0, 0, 1, 0\rangle$ would result in a forbidden output pattern. The transition probabilities for distinguishable and indistinguishable photons through a Fourier transform are highly symmetric. In the above example, it turns out to makes no difference in which mode the one distinguishable boson initially resides. Working through each of the states in P_1 and P_2 we find they all result in the same forbidden state probabilities of $\lambda_1 = \frac{4}{9}$ and $\lambda_2 = \frac{2}{3}$ respectively. Substituting into Supplementary Eq. (13) we have,

$$\mathrm{tr}\big(U^\dagger \sigma U \big(\frac{4}{9} P_1 + \frac{2}{3} P_2\big)\big) \leq p_2, \tag{14}$$

$$\Rightarrow \mathrm{tr}\big(U^\dagger \sigma U \big(P_1 + \frac{3}{2} P_2\big)\big) \leq \frac{9}{4} p_2, \tag{15}$$

where we have divided through by $\frac{4}{9}$. We can use Supplementary Eq. (15) as an upper bound on $\mathrm{tr}\big(U^\dagger \sigma U \hat{P}\big) = \mathrm{tr}\big(U^\dagger \sigma U (P_1 + P_2)\big)$, which is what we originally set out to do. We find that

$$\mathrm{tr}\big(U^\dagger \sigma U \hat{P}\big) \leq \mathrm{tr}\big(U^\dagger \sigma U (P_1 + \frac{3}{2} P_2)\big) \leq \frac{9}{4} p_2 \tag{16}$$

and thus arrive at the following final expression for the fidelity bound in Supplementary Eq. (9), to get

$$F \geq p_1 - \mathrm{tr}\big(U^\dagger \sigma U \hat{P}\big) \geq p_1 - \frac{9}{4} p_2. \tag{17}$$

Note that this step renders the lower bound loose in general, and will tend to provide a pessimistic estimate of the fidelity. Finally, we turn to the question of finite-size statistics. Many tools have been developed for this situation, and here we will make use

of a result from Supplementary Ref. [1] based on Chebyshev's inequality which states that given k independent samples and an observed fraction p_1 we can say that the 'true' probability of that outcome $\bar{p}_1$ must satisfy

$$\Pr\left[|\bar{p}_1 - p_1| \leq \delta\right] \geq \varepsilon, \;\; \delta(\varepsilon)^2 = \frac{2\Sigma}{k\log(1/\varepsilon)}, \tag{18}$$

where $0 \leq \Sigma < \infty$ is the variance of the distribution and k is the number of measurements. This can be put together to obtain Main Eq. (5) which holds with probability $\epsilon = \epsilon_1\epsilon_2$, and $\delta(\epsilon) = \delta(\epsilon_1) + \delta(\epsilon_2)$ arising from applying Supplementary Eq. (18) to the experimental observations of p_1 and p_2.

Generalization to larger systems. This certification method can be extended to arbitrarily many modes and photons. Whilst a detailed investigation of the robustness and performance of this method is beyond the scope of this work, we briefly explain how the protocol generalizes and make some comments. The scheme can be used to certify the creation the fidelity multi-partite entangled state vectors $|\psi_t\rangle = U_{\mathrm{LO}}(V)\,|\psi\rangle$ created by acting an m-mode linear optical unitary on an initial state vector $|\psi\rangle$, where the first $n \leq m$ modes are populated with indistinguishable photons as there exist forbidden states for arbitrary n. In fact, the technique can be slightly generalized further to any initial state where the photons are arranged in a periodic pattern. The generalised expression for the first measurement setting would read,

$$\mathrm{tr}\left[U^\dagger \sigma U \left(|\Psi\rangle\langle\Psi| + \sum_i \hat{P}_i\right)\right] \geq p_1 \tag{19}$$

leading to a bound

$$F \geq p_1 - \mathrm{tr}\left(U^\dagger\sigma U \sum_i \hat{P}_i\right), \tag{20}$$

where the P_i are all the different sub-spaces corresponding to the existence of different numbers bosons partitioned into different distinguishable 'species'. There can be up to n of species (i.e., one distinguishable, two distinguishable, ... , n distinguishable – if the number of species is equal to the number of photons n, then all photons are mutually distinguishable).

The second measurement setting is already described for arbitrary P_i and hence n in Supplementary Eq. (13) and, recalling that

$$\mathrm{tr}\left(U^\dagger\sigma U\sum_i \hat{P}_i\right) \leq \mathrm{tr}\left(U^\dagger\sigma U \sum_i \lambda_i \hat{P}_i\right), \forall \lambda_i \geq 1 \tag{21}$$

allows us to obtain the bound

$$F \geq p_1 - \frac{p_2}{\min_i \lambda_i} \tag{22}$$

in the general case. This scheme is manifestly efficient in the number of measurement settings (two) and also scales well in terms of the the total sample size for each probability estimate. However, to evaluate the bound we naturally need to know the value of the λ_i and also the set of forbidden states to determine p_2. These calculations are a one-off cost in the sense that it need only be performed once ahead of time for any value of n and can then be used to certify all states in the corresponding class. In this sense, it is not counted in the scaling cost of the protocol, nevertheless it is a non-trivial overhead and we discuss the calculation in some more detail.

To explain things further we briefly recall some notation and results from Supplementary Refs. [4–6]. Let $r = (r_1, r_2, \ldots, r_m)$ and $s = (s_1, s_2, \ldots, s_m)$ be the input and output mode occupation list with $\sum_i s_i = \sum_i r_i = n$. In our case, we have $m = 4$ modes and $n = 3$ photons. Our input mode occupation has been $r = (1, 1, 1, 0)$. A useful alternative notation for the mode occupation list is the *mode assignment list* $d(q)$, which is structured in terms of photons rather than modes. Its entries represent the photons and the numerical value indicates the mode that is being occupied by that photon (the list has as many entries as there are photons as opposed to as many entries as there are modes). For example, the mode occupation list $r = (2, 0, 0, 1)$ becomes $d(r) = (1, 1, 4)$ (the first and second photon being in mode one and the third photon in mode four). The general expression for the mode assignment list given a mode occupation list q reads

$$d(q) = \bigoplus_{j=1}^{m}\bigoplus_{k=1}^{q_j}(j) = (\underbrace{1,\ldots,1}_{q_1}, \underbrace{2,\ldots,2}_{q_2}, \ldots, \underbrace{m,\ldots,m}_{q_m}). \tag{23}$$

For bosons, the transition probabilities through a Fourier transform are proportional to the permanent of an $n \times n$ sub-matrix M of the $m \times m$ Fourier matrix V_n^{Four}. With this notation of mode assignment lists, we can neatly express the transition probabilities as

$$P(r, s, V_n^{\mathrm{Four}}) = \frac{|\mathrm{perm}(M)|^2}{\prod_j r_j! s_j!} \tag{24}$$

for the case of indistinguishable photons and

$$P(r, s, V_n^{\mathrm{Four}}) = \frac{\mathrm{perm}(|M|^2)}{\prod_{j=1}^{n} s_j!} \tag{25}$$

for distinguishable ones. The $n \times n$ matrix constructed from V_n^{Four}, referred to as M, is defined as

$$M_{j,k} := (V_n^{\mathrm{Four}})_{d_j(r), d_k(s)} \tag{26}$$

where $d_j(r)$ is the jth element of the mode assignment list $d(r)$ and the elements of V_n^{Four} are given in Supplementary Eq. (10).

The forbidden patterns can efficiently be calculated as the strictly suppressed output states of the indistinguishable case with respect to the chosen Fourier transform V_n^{Four}. More precisely, in Supplementary Ref. [4] it has been shown that, for a given (potentially p-periodic) initial state r, final states s are suppressed through quantum interference when the criterion

$$\mathrm{mod}\left(p \sum_{j=1}^{N} d_j(s), n\right) \neq 0 \tag{27}$$

holds, i.e., if the above criterion holds, then the transition probability $P(r, s, V_n^{\mathrm{Four}})$ in Supplementary Eq. (24) vanishes. Having found the suppressed (i.e., forbidden) patterns, one can then go ahead and compute the λ_i (probability that states in the various P_i would result in a forbidden state). As explained above, for a given P_i one needs to consider the probabilities that the populations of the distinguishable species can combine to result in a forbidden state. Strictly speaking, to evaluate Supplementary Eq. (22) we only need the value of the smallest λ_i. Based on preliminary investigations we conjecture that the case of $n-1$ indistinguishable bosons and 1 distinguishable boson is the minimal case. If it were necessary to check all of the λ_i, it is not trivial to determine how many calculations this would entail as it corresponds to the problem of placing n indistinguishable objects in k indistinguishable boxes (the bosons in each species are of course mutually distinguishable, here we are using indistinguishable in the sense that the situation that the arrangement with, say, 3 bosons in species 1 and 2 bosons in species 2 is, for our purposes, equivalent to 3 bosons in species 2 and 2 bosons in species 1) for which there is no compact form. A crude upper bound for a given n and an number of species would be to count the number of ways of placing n objects in k *distinguishable* boxes which would upper bound the number of λ_i to be calculated via $\sum_{j=2}^{n} \binom{n-1}{j-1}$. Even if our conjecture is true, calculating a single λ_i would still involve evaluating the transition probability (and hence matrix permanent) for $n-1$ bosons, which is classically hard in general. Nevertheless, to our knowledge the hardness for the specific case of a Fourier transform remains open, which leaves the total complexity of this calculation unclear for the present.

SUPPLEMENTARY NOTE 2 – GAUSSIFICATION

For completeness, in this subsection we recall some previous work on Gaussification and present some numerical results illustrating approximate Gaussification for the systems considered in this work. For non-interacting quadratic bosonic Hamiltonians, such as describe linear quantum optics experiments of the kind considered here, the mechanisms for equilibration have been well studied [7–11]. In particular, it has been rigorously shown that systems will tend to 'Gaussify', meaning that after a sufficiently long enough time has elapsed, any subsystem (or even a block of subsystems) will converge to Gaussian, maximum entropy states and remain there. For finite number of modes and bosons we will never find the subsystems in perfectly Gaussian state, nor will they remain in such a state indefinitely. Instead the system will approximately Gaussify with the closeness of the approximation depending upon the system size.

More formally, consider a state vector $|\psi\rangle \in \mathcal{H}_U = \mathcal{H}_S \otimes \mathcal{H}_E$ of the entire 'universe' of our experiment which comprises m modes/sites which we can think of as a system S and environment E with given second moments in the creation annihilation operators $\langle \hat{b}_i^\dagger \hat{b}_j \rangle >$ (i.e., the photon occupancy). Define a reduced state of a subsystem $\varrho_S = \mathrm{tr}_E \{|\psi\rangle \langle\psi|\}$. In our work, we have been focusing on the state of this subsystem and considering just a single mode, but one could also think of a larger subsystem. We are then interested in the dynamics as a function of time and system size. Taking the thermodynamic limit will involve fixing ratio of photons to modes (n/m) and then considering the limit $m \to \infty$. In that case we want to know if ϱ_S will eventually equilibrate to ϱ_S^{mc}, the micro-canonical state on the subsystem given the constraints on the second moments. In this limit, for fixed second moments, the maximum entropy state is a Gaussian state $\varrho_S^{\mathrm{me}} = \varrho_G$. Approximate Gaussification can then be expressed as the condition that, for any S and any $\varepsilon > 0$ there exists an m and relaxation and recurrence times t_{Rec} and t_{Relax} , such that

$$\|\varrho_S(t) - \varrho_G\|_{\mathrm{tr}} < \varepsilon \quad \text{for } t \in [t_{\mathrm{Relax}}\,, t_{\mathrm{Rec}}]\,. \tag{28}$$

Even for the modest system sizes at play in this work, it is still possible to observe substantial Gaussification. In Supplementary Fig. 2 we numerically calculate the Wigner functions for each of the 4 modes for the initial input states at $t = 0$ and an evolved state at $t = 1$ for both the hopping Hamiltonian and one of the Haar-random, long-range Hamiltonians. Whilst the initial state exhibits substantial non-Gaussianity and Wigner negativity for the modes initially occupied with a single photon, after evolution all modes appear as approximately Gaussian with additional modulation caused by finite-size effects and all Wigner negativity has vanished. It is interesting to note that mode 4, which has initially been in a perfectly Gaussian vacuum state, is technically less Gaussian after evolution. Nevertheless, the system still exemplifies the phenomena described in Supplementary Eq. (28), as Gaussification is a claim that must hold for *all* subsystems and not just one. After evolution the condition that all modes can be well-approximated by a Gaussian is satisfied, whereas it is radically violated by the initial input state.

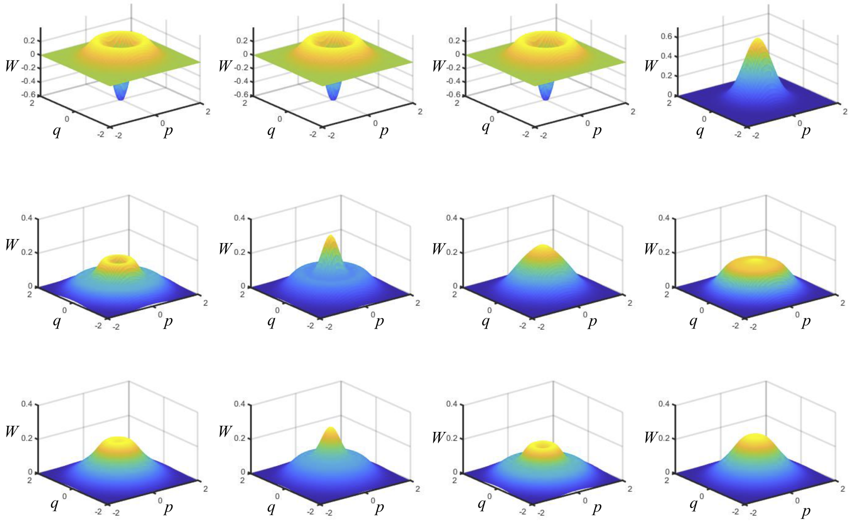

Supplementary Figure 2. **Approximate Gaussification.** Theoretical Wigner functions of each mode (columns) for the initial input state at $t = 0$ (top row) and the evolved state ($t = 1$) for the hopping Hamiltonian (middle row) and a long range Hamiltonian (bottom row). The Wigner quasiprobability W is plotted as a function of dimensionless continuous momentum p and position q eigenvalues.

SUPPLEMENTARY NOTE 3 – INDISTINGUISHABLE PHOTON QUALITY

To characterize the quality of our prepared indistinguishable $|1, 1, 1, 0\rangle$ input state vector, we have measured HOM-dips (see Supplementary Fig. 3), using our processor as a beam splitter between pairs of modes. We find HOM visibilities of $V_{s_1,i_2} = |\langle\psi_{s_1}|\psi_{i_2}\rangle|^2 = 89.1\%$, $V_{s_1,s_2} = |\langle\psi_{s_1}|\psi_{s_2}\rangle|^2 = 92.3\%$, and $V_{s_2,i_2} = |\langle\psi_{s_2}|\psi_{i_2}\rangle|^2 = 94.3\%$.

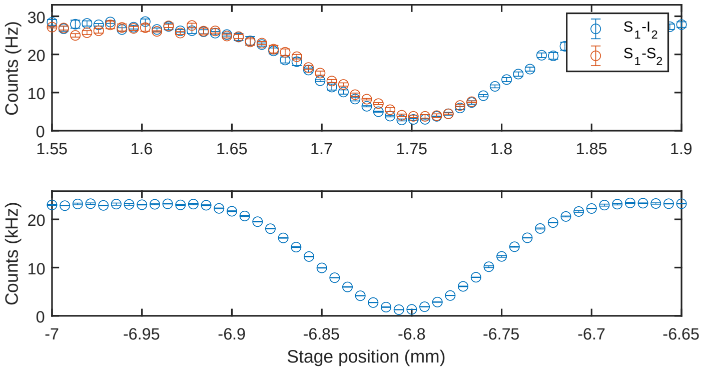

Supplementary Figure 3. **Source pre-characterization.** Top panel: typical intermodal HOM dips result in visibilities of 0.89 (signal crystal 1 s_1 - idler crystal 2 i_2) and 0.92 (signal crystal 1 s_1 - signal crystal 2 s_2) for photons generated in different crystals. Bottom panel: the HOM visibility of a pair of photons generated by crystal 2 (signal crystal 2 s_2 - idler crystal 2 i_2) is typically around 0.94. Error bar represents the standard error based on 5 measurements.

SUPPLEMENTARY NOTE 4 – QUANTUM PHOTONIC PROCESSOR OPERATION FIDELITY

To test the fidelity of the implemented optical transformations, we perform a calibration experiment. For this, classical CW light from a 1550 nm super luminescent diode (Thorlabs S5FC1005P) is injected into the input modes and an array of calibrated photodiodes (Thorlabs FGa01FC) are used to detect the output signal. The amplitude fidelities are defined as $F := \frac{1}{n}\mathrm{Tr}(|U^{\dagger}_{\mathrm{set}}||U_{\mathrm{get}}|)$, where U_{get} denotes observed transfer matrix, U_{set} is the target transfer matrix and the absolute signs indicate the element-wise absolute value of the matrix elements, and $n = 12$ modes is the size of the transfer matrices. For a set of 150 random permutation matrices, a value of $F = 0.992 \pm 0.002$ is found, whereas for a set of 100 Haar-random matrices we find $F = 0.979 \pm 0.01$. The full histograms of these measurements are shown in Supplementary Fig. 4.

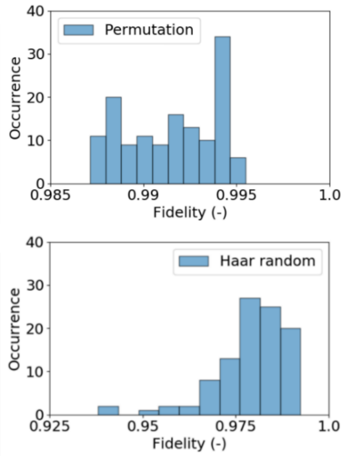

Supplementary Figure 4. **Matrix fidelities.** Histogram of amplitude fidelities over two families of random matrices. Top panel: fidelity of random permutation matrices. Bottom panel: fidelity of random matrices.

SUPPLEMENTARY NOTE 5 – PHOTON DETECTOR BLINDING

Although post-selection on heralded three-photon events allows for extracting events based on the input state vectors $|\psi\rangle = |1, 1, 1, 0\rangle$, other, unwanted states are frequently produced because of the probabilistic nature of SPDC sources, e.g., when one source produces a photon pair but the other one does not. Unwanted by-product states cause detector blinding, which in combination with imperfect matrix fidelities biases observed photon statistics. This effect is illustrated in Supplementary Fig. 5 for the identity matrix transformation. Therefore, the observed photon statistics is dependent on used pump power levels. Especially photon statistics for unitary matrix transformations close to the identity matrix transformation are affected as can be seen in Main Fig. 3, as detector blinding is more likely due to most of the light being directed to a limited set of detectors.

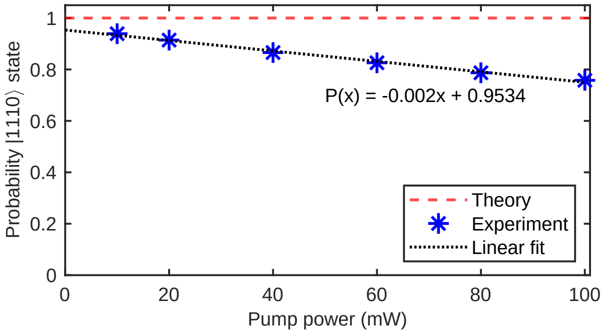

Supplementary Figure 5. **Detector blinding as function of pump power.** The horizontal axis shows the pump power, the vertical axis shows the probability of observing the outcome $\mu = (1, 1, 1, 0)$ when the identity matrix is dialled on chip, which is the only expected outcome in this case. For higher pump-power levels, the detrimental effect of by-products is increased. In case of perfect realized unitary fidelity, measurement probability is independent of pump power. Empirically, we find this effect is well described by $P(\mu) = -0.0020 \cdot P_{\text{pump}} + 0.9534$ for $\mu = (1, 1, 1, 0)$ with pump power P_{pump} in mW. Error (standard deviation based on Poisson statistics) is within symbol size.

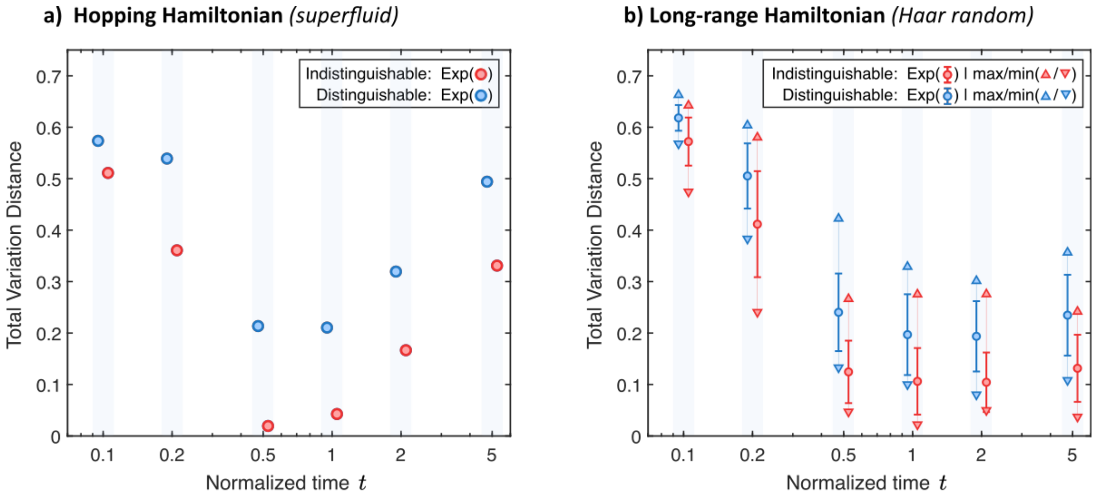

Supplementary Figure 6. **Total variational distance (TVD) to the canonical thermal state as a function of normalized time t.** The red and blue symbols correspond to indistinguishable and distinguishable photons, respectively. The left panel (a) shows the TVD for the six experimentally (Exp) simulated time steps on the hopping Hamiltonian. The right panel (b) shows the TVD for the six time steps experimentally (Exp) simulated for the set of long-range Hamiltonians. In this panel, the average, minimum (min), maximum (max) TVD, as well as the standard deviation (error bar) in the TVD are shown.

SUPPLEMENTARY NOTE 6 – DETAILED EXPERIMENTAL RESULTS

In this section, we provide additional information on the results of our experiments. To better illustrate the convergence to Main Eq. (4), the total variation distance between the experimental data and the canonical probability density function (from the first panel of Main Fig. 3a and 3b) is shown in Supplementary Fig. 6. Furthermore, we consider the achievable fidelity bound as a function of measurement time. The certification fidelity only becomes meaningful when the probability of error $1-\epsilon$ is sufficiently small. This requires prolonged measurement times to accumulate sufficient statistics. Moreover, detector blinding (see Supplementary Fig. 5) limits the average pump power for the fidelity certification measurements to only $5\,\mathrm{mW}$ per crystal. Such low pump power results in a fourfold coincidences rate of around $4\,\mathrm{Hz}$. To prevent a bias caused by long term drift, all certified time steps are measured 'interleaved', i.e., each certification measurement is repeated throughout multiple times for short run time.

Supplementary Figs. 7 and 8 depict how the certification fidelities converge when the total measurement time is increased. Each data point is the result of a 20 minute measurement per certification step, i.e., 40 minutes per data point. The horizontal axis is linearized as $1/\sqrt{T}$, where T is the measurement time in hours. Empirically, we find that the certified fidelity decreases linearly on this scale (i.e., increases linearly with $-1/\sqrt{T}$). This is consistent with the independent nature of the separate experimental runs. The red horizontal dashed lines are the bi-partition (spatial mode 1 vs spatial modes 2,3,4) fidelities required to certify entanglement. Finally, the blue solid line is a linear fit through the data points. The fit is extrapolated to 100 hours of measurement time. The number at the end (left) of the fit is the corresponding maximum fidelity expected based on this extrapolation.

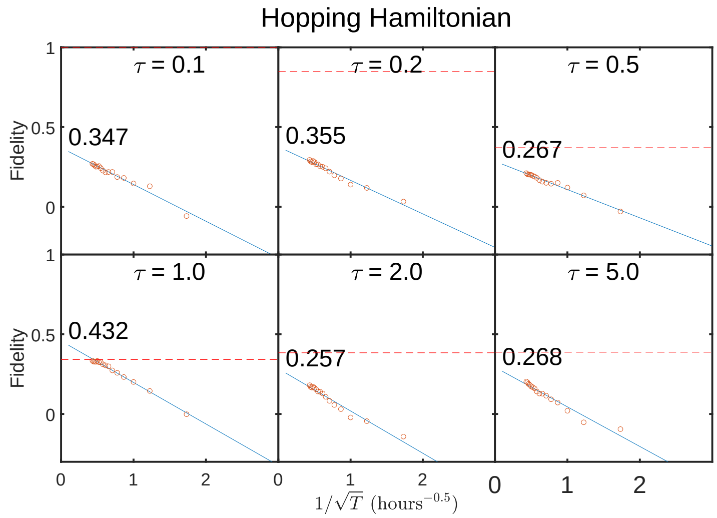

Supplementary Figure 7. **Convergence of certification statistics**. Superfluid/short-range order system certification as a function of measurement time for all 6 normalized time steps τ, for confidence level $\epsilon = 0.9$. T indicates the measurement time.

Supplementary Fig. 7 shows the convergence of the certification fidelity for the non-interacting Bose-Hubbard, or superfluid, Hamiltonian. Each panel corresponds with one of the six simulated time steps. There are a total of 16 batches for each time step, which is almost sufficient to certify $\tau = 1.0$ against the bi-partition with $\epsilon = 0.9$. Therefore, we included 10 more batches measured under similar conditions to increase the fidelity for $\tau = 1.0$ from $F = 0.335$ to $F = 0.359$. Furthermore, the extrapolations indicate that longer measurement times are not going to certify the remaining simulated time steps.

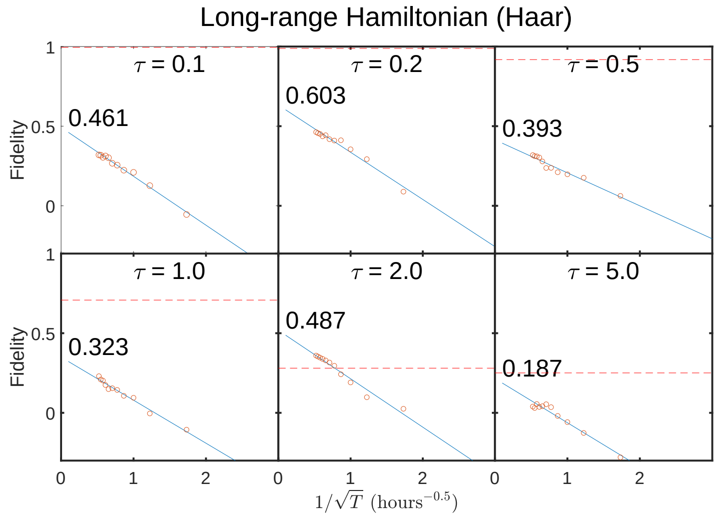

Supplementary Figure 8. **Convergence of certification statistics**. Certification of the first long-range order/Haar random Hamiltonian system as a function of time for all 6 normalized time steps τ, for confidence level $\epsilon = 0.9$. T indicates the measurement time.

Similarly, the first long-range Haar-random system's certification converges as shown in Supplementary Fig. 8. There are a total of 11 batches for each time step. Here, the simulated time step of $\tau = 2.0$ is clearly certified against the bi-partition. Unfortunately, the other time steps will not be able to reach the required certification fidelities when the measurement time is increased.

---

[1] L. Aolita, C. Gogolin, M. Kliesch, and J. Eisert, Nature Comm. **6**, 8498 (2015).
[2] N. Friis, G. Vitagliano, M. Malik, and M. Huber, Nature Rev. Phys. **1**, 72 (2019).
[3] C. K. Hong, Z. Y. Ou, and L. Mandel, Phys. Rev. Lett. **59**, 2044 (1987).
[4] M. C. Tichy, M. Tiersch, F. de Melo, F. Mintert, and A. Buchleitner, Phys. Rev. Lett. **104**, 220405 (2010).
[5] M. C. Tichy, M. Tiersch, F. Mintert, and A. Buchleitner, New J. Phys. **14**, 093015 (2012).
[6] M. C. Tichy, K. Mayer, A. Buchleitner, and K. Mølmer, Phys. Rev. Lett. **113**, 020502 (2014).
[7] M. Cramer, C. M. Dawson, J. Eisert, and T. J. Osborne, Phys. Rev. Lett. **100**, 030602 (2008).
[8] M. Cramer and J. Eisert, New J. Phys. **12**, 055020 (2010).
[9] M. Gluza, J. Eisert, and T. Farrelly, SciPost Phys. **7**, 038 (2019).
[10] T. Schweigler, M. Gluza, M. Tajik, S. Sotiriadis, F. Cataldini, S.-C. Ji, F. S. Møller, J. Sabino, B. Rauer, J. Eisert, and J. Schmiedmayer, Nature Phys. **17**, 559 (2021).
[11] T. Monnai, S. Morodome, and K. Yuasa, Phys. Rev. E **100**, 022105 (2019).
